# Advancements in Multiple Myeloma Research: High-Throughput Sequencing Technologies, Omics, and the Role of Artificial Intelligence

**DOI:** 10.3390/biology13110923

**Published:** 2024-11-13

**Authors:** Alejandra Gutiérrez-González, Irene Del Hierro, Ariel Ernesto Cariaga-Martínez

**Affiliations:** DS-OMICS—Data Science and Omics, AI-Driven Biomedicine Group, Universidad Alfonso X el Sabio, 28619 Villanueva de la Cañada, Spain; alegugon@uax.es (A.G.-G.); idelhgar@uax.es (I.D.H.)

**Keywords:** multiple myeloma, biomarkers, diagnostics, prognosis, next-generation sequencing, third-generation sequencing, omics, artificial intelligence, machine learning, deep learning

## Abstract

Multiple myeloma is a complex and challenging type of blood cancer that affects plasma cells in the bone marrow. In recent years, the development of advanced research techniques, such as omics approaches—which involve studying large sets of biological data like genes and proteins—and high-throughput sequencing technologies, has allowed researchers to analyze vast amounts of genetic information rapidly and gain new insights into the disease. Additionally, the advent of artificial intelligence tools has accelerated data analysis, enabling more accurate predictions and improved treatment strategies. This review aims to highlight recent research advances in multiple myeloma made possible by these novel techniques and to provide guidance for researchers seeking effective approaches in this field.

## Simple Summary

Multiple myeloma is a complex blood cancer that targets plasma cells. Recent advances in omics technologies, high-throughput sequencing, and artificial intelligence have enhanced data analysis, leading to improved predictions and treatment strategies. This review highlights recent progress in these research areas and provides direction for future studies.

## 1. Introduction

Multiple myeloma (MM) is the second most common hematologic malignancy after non-Hodgkin’s lymphoma, accounting for approximately 1.8% of all new cancer cases [1]. This cancer is characterized by the clonal proliferation of malignant plasma cells (PCs) in the bone marrow (BM) and exhibits a complex genetic landscape that enables the classification of patients into subgroups with differing prognoses, treatment responses, and levels of drug resistance [2,3]. Common symptoms of MM include bone damage, anemia, impaired kidney and immune function, and hypercalcemia, along with destructive bone lesions and the production of abnormal monoclonal immunoglobulins [4,5]. With the use of approved modern drug combinations, a complete response can be achieved in most patients. However, relapses still occur, indicating the persistence of a small but clinically significant subset of myeloma cells, known as minimal residual disease (MRD) [6]. Patients who experience these relapses may develop what is known as relapsed or refractory multiple myeloma (RRMM), in which their disease no longer responds to previous treatments and requires new lines of therapy [7,8]. In general, MM median survival is approximately six years [4].

The diagnosis of MM is highly complex due to the presence of various asymptomatic precursor stages, such as monoclonal gammopathy of undetermined significance (MGUS) and smoldering multiple myeloma (SMM). MGUS occurs in 3% of the population over 50 years old and can progress to MM or an associated malignant disorder at a rate of 1% per year. Additionally, some patients may develop the asymptomatic intermediate stage known as SMM [9]. Additionally, other hematologic malignancies, including primary amyloidosis, Waldenström macroglobulinemia, and plasma cell leukemia (PCL), share overlapping characteristics with MM, making precise differential diagnosis crucial for timely and effective treatment [10]. Moreover, the absence of symptoms in the earliest stages of the disease often leads to MM diagnosis in hospital emergency settings [11].

Once the diagnosis is established, biomarker identification helps determine the genetic heterogeneity driving disease progression and correlates with the development of the various stages of MM [12,13]. For example, evolution from MGUS to MM is associated with the expression of oncogenes MYC and RAS [14,15], as well as chromosome deletion and DNA hypomethylation [16,17]. Other common genetic alterations include chromosomal translocations, gains and losses of chromosomes that target the cyclin D (CCND) family, immunoglobulin heavy chain (IGH) translocations—most commonly t(11;14)(q13;q32), detected in 20% of MM patients—and alterations in the immunoglobulin lambda (IgL) gene locus, reported in 10% of MM patients [18,19] (Table 1).

The primary traditional techniques to detect these biomarkers include BM biopsy, serum analysis, 24 h urine analysis, metaphase karyotyping, fluorescence in situ hybridization (FISH), imaging, assessment of monoclonal protein (M protein), identification of abnormal immunoglobulins, measurement of free light chains in the serum and urine of MM patients, as well as detection of chromosomal abnormalities and osteolytic bone lesions [13,20]. Techniques such as fluorescence in situ hybridization (FISH) and single nucleotide polymorphism (SNP) microarrays allow for the detection of chromosomal aberrations but have certain limitations. For example, while FISH is highly accurate in identifying recurrent aberrations, it is restricted to the targets of the selected probes, preventing a complete cytogenetic characterization. SNP microarrays, on the other hand, are used to summarize copy number changes in MM but often rely on FISH results to detect IGH translocations [2]. Moreover, FISH and SNP microarrays cannot detect somatic point mutations [12,21]. Additionally, these methodologies are labor-intensive, have limited genomic resolution, and require large quantities of bone marrow material, which is often inadequate due to the low mitotic activity and low percentage of plasma cells in the bone marrow in MM patients [13].

With the availability of various cytogenetic and molecular techniques, clinical guidelines have been developed to distinguish between the early and advanced stages of MM. The International Myeloma Working Group (IMWG) has presented diagnostic benchmarks for MM and its differential phases [22]. Additionally, two systems, the Durie-Salmon PLUS System (DSS) and the International Staging System (ISS), have been established to assess MM progression, patient survival rates, and treatment protocols [23,24]. It is important to note that these guidelines depend on the sensitivity and reliability of biomarker detection, which can be complex and prone to misdiagnosis or inaccurate staging. For instance, M-protein is undetectable in 18% of MM cases using serum protein electrophoresis, and 3% of patients lack reportable traces [25,26]. To address these limitations, researchers are now focused on discovering novel biomarkers to improve diagnostic accuracy, including angiogenesis markers, microRNAs, telomeres and telomerase activity, extracellular matrix (ECM) proteins, circulating tumor cells and DNA, and genomic, proteomic, and immunologic markers. Advanced methodologies for detecting these biomarkers include multiparameter flow cytometry (MPC), next-generation sequencing (NGS), liquid or blood biopsy, and allele-specific oligonucleotide (ASO)-qPCR [13,20].

The complexity of MM poses significant challenges in diagnosis, prognosis, and treatment, highlighting the need for innovative methods to improve patient outcomes. In this regard, artificial intelligence (AI) and high-throughput sequencing technologies (NGS and TGS), along with recent advancements in omics, represent transformative tools in modern medicine with the potential to revolutionize cancer care. Through machine learning (ML) and deep learning (DL) algorithms, AI has shown remarkable potential in analyzing large datasets, identifying patterns, and making predictions that enhance clinical decision-making [27]. Likewise, NGS enables comprehensive genomic profiling, allowing for the identification of genetic mutations and alterations at an unprecedented level of resolution [28].

This review aims to provide a comprehensive overview of the most significant research conducted in recent years on the application of AI and NGS in the field of MM, highlighting the advancements and potential of these technologies in deepening our understanding of this complex disease. It will synthesize the current state of knowledge, identify emerging trends, and discuss the implications of AI and NGS for future research and clinical practice in MM.

## 2. Overall Aspects of High-Throughput Sequencing

Personalized medicine is a highly promising approach aimed at achieving greater treatment efficacy and reducing side effects in cancer care. This strategy is closely tied to the identification of disease-related mutations and genetic variations, which can be effectively accomplished through high-throughput sequencing. Leading this group of technologies is next-generation sequencing (NGS), or second-generation sequencing, which has evolved rapidly over the past 15 years from classical methods like Sanger and Maxam–Gilbert sequencing. Although the Sanger method remains valuable for research purposes, it has largely been replaced by NGS due to its lower costs and much higher capacity.

NGS methods are broadly categorized by three main companies and their associated technologies. The first technology would be Pyrosequencing (QIAGEN, Hilden, Germany) primarily using 454 sequencers, though this technology is now largely outdated. The other two sequencing technologies are Ion Torrent (Thermo-Fisher, Waltham, USA) and Illumina (Illumina, San Diego, USA). While both are widely used today, Illumina’s technology has become the leading NGS platform, significantly surpassing traditional Sanger sequencing in terms of efficiency, accuracy, and speed. Illumina’s technology can read up to 300 bps and perform paired-end sequencing, which is essential for identifying chimeric DNA molecules [29].

Different inputs of DNA and the machines employed result in three main NGS strategies: whole-genome sequencing (WGS), whole-exome sequencing (WES or coding genome), and targeted panels sequencing (TS, sequencing of limited areas of interest). The most complex and complete strategy would be WGS, which allows the identification of coding and non-coding mutations, INDELs, aneuploidies, CNAs, structural rearrangements and signatures, as well as intergenic, intronic, and untranslated regions (UTRs), promoters, regulatory elements, and repetitive regions, all being areas capable of harboring multiple cancer-driving mutations. WES would give information about the coding genome, which corresponds only to 1–2% of the total genome, thus retrieving information on coding mutations, INDELs, aneuploidies, and CNAs. The final one, the TS, would retrieve the same information as the WES but for limited areas. TS is performed to overcome the limitations of WES and WGS by combining sensitivity and accuracy with time efficiency and cost reductions [30].

The larger footprints obtained by WGS are significantly more expensive and slower and have high costs in the downstream analyses. On the bright side, WGS does not require prior knowledge and captures the full spectrum of structural variations and mutations that may impact the development of the disease [31,32,33]. WES is primarily used to identify critical short genomic variants in cancer [34,35] but it analyzes only the coding portion of the genome, thus limiting its ability to detect translocations and CNAs [36]. Nevertheless, despite covering only a small percentage of the genome, coding regions contain over 85% of disease-related mutations in many types of cancer [37,38]. Targeted panels, on the other hand, are the most cost-effective approach for detecting and studying MM in clinical settings, as they identify mutations, CNAs, and all known immunoglobulin rearrangements. The limitation of this strategy is that it requires prior knowledge of the mutations to target, which could result in missing some significant aberrations. Still, NGS is emerging as a powerful diagnostic and prognostic tool in the clinic due to its flexibility, reduced costs, and compatibility with other techniques, such as CRISPR-based approaches [39] enabling the development of custom NGS panels like MSK-IMPACT^®^ or FoundationOne^®^ CDx [40,41,42,43,44,45,46,47,48].

In summary, WGS offers greater accuracy in capturing comprehensive whole-genome information, including CNAs, and produces less bias when identifying non-reference alleles. However, WES and TS are often preferred due to their lower cost—which enables the inclusion of larger patient cohorts—faster turnaround times, and simpler data interpretation [49,50]. Depending on the nature of the study—whether research-based or diagnostic—the available technology, budget, and sample volume, different methods should be employed [51].

### 2.1. NGS in the Study of MM

Despite the numerous advantages NGS offers, its incorporation into clinical practice for MM diagnosis has been slow and met with resistance. This hesitation is partly due to the significant heterogeneity of MM and the lack of consensus on how, or even if, NGS should be used to redefine high-risk disease [51,52,53]. For example, in acute myeloid leukemia, NGS has been introduced in a quicker way due to the lower complexity of the disease and, thus, the obtention of clearer translational results [54,55,56,57]. The primary reason for this slow incorporation is the persistent knowledge gap between computational and experimental fields. The computational field is often perceived as slow, complex, and costly, with results that are challenging to interpret and reproduce. However, a targeted NGS panel can provide a more precise and efficient assessment of various mutations and genomic alterations compared to FISH, karyotyping, or SNP microarrays [40].

Against all reluctance, NGS has proved itself to be extremely useful in the study and diagnosis of MM, aiding in the discovery of several novel genomic markers, such as genetic loci rs12521798 and rs17748074 linked to bortezomib-induced peripheral neuropathy in MM patients [58]; glutathione-s-transferase (GST) polymorphisms and tumor necrosis factor-α (TNF-α) associated with the survival in MM [59]; polymorphism rs4240803 of SLC7A45 linked to a better response in MM patients with melphalan based therapy [60]; and hypodiploidy or hyperploidy associated with a poor prognosis in MM [61,62]. Additionally, it is very interesting to highlight the results of the CoMMpass trial (https://themmrf.org/finding-a-cure/personalized-treatment-approaches/; accessed on 11 November 2024), which has revealed a complex landscape of structural variations as well as recurrent somatic mutations in the MM in several studies [19,63].

To achieve the most effective NGS techniques for MM and perform one single assay, several groups have developed TS assays specific to genomic aberrations in the disease [15,36,40,43,48,64,65]. Specifically, Yellapantula and collaborators published the first large-scale head-to-head comparison of standard FISH and SNP microarray with a custom capture NGS assay. Results of extremely high concordance led to the conclusion that NGS can be employed as a novel strategy to replace the current standard of care techniques, like FISH or SNP microarrays.

Not only can DNA material be analyzed by NGS—referred to as DNAseq or DNA sequencing—but RNA material can also be studied, with traditional gene expression profiling now replaced by RNA sequencing (RNAseq). RNAseq provides a comprehensive view of gene expression and enables comparisons between different conditions, such as mutated versus wild type, to assess expression differences and their potential effects on metabolic pathways and phenotype. Combined approaches using DNAseq and RNAseq have proven highly effective; for instance, WGS-RNAseq combinations have been used to analyze heterogenous clonal structures in patients, determine molecular profiles during disease progression and in response to treatment, and identify novel therapeutic targets for MM, such as BCMA and SLAMF7 [32,66]. In addition, low-pass WGS and RNAseq were employed in liquid biopsies of MM and other cancers to assess the circulating tumor cells and circulating free DNA [67,68,69,70,71]. These latter combinatory strategies are very promising as they require only one blood sample for the genome profiling, while the standard profiling techniques require a BM biopsy. A recent study created a portal for alternative splicing events for each MM subgroup, thanks to the obtained NGS results [72].

In addition to genomic and genetic landscapes, the rapidly expanding field of epigenetics also plays a crucial role, with sequencing technologies aiding in the analysis of DNA methylation, histone modifications, and non-coding RNA expression. In MM, DNA has been observed to be globally hypomethylated, except at the promoters of tumor suppressor genes, where it is instead hypermethylated [73]. Methylation levels increase from MGUS to MM, revealing novel biomarkers associated with poor prognosis [74,75,76,77,78,79]. Regarding histones, overexpression of histone deacetylases and other factors like NSD2 and EZH2 have been associated with poor prognosis in MM [53,73]. Last, in epigenetics, we encounter that non-coding RNAs, specifically microRNAs (miR), play a role in MM, with their signatures being different between normal and tumoral cells, they show increased deregulation in MM compared to MGUs [73], and they have a clear impact on tumor initiation, progression, and metastasis [80,81]. Although their presence has been typically associated with poor outcomes, their early detection has improved survival prediction in MM patients [82].

### 2.2. Insights and Advances in the Different Stages of MM

In every stage of the MM, NGS has been employed to unveil some knowledge about MM, as can be seen in Table 2. To start with the newly diagnosed multiple myeloma (NDMM) patients, NGS allowed the identification of several risk factors such as amp(1q) [83], del(1p) and del(12p) [84], del(17p) related to rare states of hypodiploidy or hyperaploidy [61,85,86], and immunoglobulin lambda translocations related to a poor prognosis [19]. Additionally, a reported rare case of Gaucher-like cristal-storing histiocytosis (CSH), associated with kappa chain deposits and unusual amino acid substitutions in the variable region of the kappa chain, was associated with a poor outcome of the disease [87], although kappa alterations have also been related to myeloma in patients with trisomies [88]. Mutations like EGR1 and IRF4 were discovered to be related to a good prognosis, whilst ZFHX4 led to a bad one in the Myeloma XI UK trial [36]. Studies of the whole mutational spectrum of NDMM correlated hypermutated states with worse prognosis [17,89], as well as the extent of cytogenetic lesions that are correlated with prognosis inversely proportional to their number [43,90,91]. Interestingly, APOBEC contribution for mutational signatures amplifies a bad prognosis no matter the number of mutations or the cytogenetic group [3,31,92]. Altogether, it is being revealed that survival in NDMM is probably influenced by an increasing genomic complexity rather than the presence or absence of genetic lesions. However, this hypothesis should be evaluated as novel risk factors continue to appear in the MM diagnostic scenario [93].

Progression to MGUS has been defined by myeloma-defining genomic events obtained thanks to several low-pass WGS analysis [94,95,96], which has led to the idea of redefining the stages of myeloma from low-risk MGUS, intermediate-risk MGUS, high-risk MGUS, low-risk SMM, intermediate-risk SMM, high-risk SMM, and MM to monoclonal gammopathy, early multiple myeloma, and multiple myeloma [97]. However, as the state of the art continues with the former classification, we will continue with it in this review.

In the intermediate stage or SMM, combinatory DNA and RNA strategies have revealed that asymptomatic stages carry a globally lower number of mutations than NDMM [57,98]. In other studies, genomic changes were discovered to be the cause of the spontaneous evolution of cancer cells, but in other scenarios, environmental factors or the accumulative mutation burden were the cause [3,66,99]. WGS has been employed to analyze rearrangements in SMM, discovering that only IGH-MYC rearrangements confer a high risk of SMM progression [100] and complex rearrangements were discovered to be equally present in SMM than in MM but with a lower cancer cell fraction in the first [3]. NGS was also the cause of the discovery that high-risk SMM genomes were more like NDMM due to the timing and activity of mutational processes like the aberrant activity of the APOBEC family of DNA deaminases [3,31,92]. Finally, chr(8p) deletions, DNA tumor fractions, and number of alterations have been suggested to have clinical relevance in the progression from SMM to MM [46].

In the final stage of MM, RRMM much less is known due to the high heterogeneity of subtypes that show an increased number of mutations, abnormalities in the copy numbers, complex rearrangements, and novel mutational signatures [17,31,44,63,101]. Targeted sequencing was employed to detect mutations conferring resistance to IMiDs and PIs [41,102], but most of the mutations were subclonal, which implies that clinical impact is still in check. Recently, a study suggested that co-occurrence of 1q21 gain/amplification and MAPK mutations are crucial mutational events in the development of extramedullary multiple myeloma, an aggressive form of MM [103].

**Table 2:** High-throughput discoveries in the different stages of MM.

MM Stage	Key Findings [Reference]	Related Prognosis
NDMM	Amp(1q) [83]; del(1p) and del(12p) [84]; del (17p) and hypodiploidy/hyperploidy [61,85,86]; lambda translocations [19]; kappa alterations [87,88]; mutation ZFHX4 [36]; general hypermutation [17,89], APOBEC alterations [3,31,92]	Risk factor and poor prognosis
NDMM	Mutations EGR1 and IRF4 [36]	Good prognosis
MGUS	Global state of higher mutation rate than NDMM and lower mutation rate than SMM [7,94]; genomic-defining events that lead to successive stages [94,95,96,97]	Bad prognosis when reaching high-risk MGUS from low-risk and intermediate-risk stages
SMM	General higher mutation rate than MGUS [57,90,91,92,93]; genomic changes, environmental factors, and mutational burden [3,66,99]; IGH-MYC [100]; complex rearrangements with lower cancer cell ratio than MM [3]; chr(8p) deletions [46]	Bad prognosis
MM and RRMM	High rate of mutations, abnormalities in the copy numbers, rearrangements, and novel signatures [17,31,44,63,101]; co-occurrence of 1q21 gain/amplification and MAPK mutations [103]	Bad prognosis and possible development of extramedullary MM

### 2.3. Third-Generation Sequencing Advances and Current State

As mentioned above, the importance and utility of NGS in the study and diagnosis of MM have been demonstrated in multiple studies, yielding sensitive, specific results with verified reproducibility, as evidenced by the EuroClonality-NGS Working Group [104,105].

Recent advances in sequencing have led to the emergence of third-generation sequencing (TGS), with key proponents being Oxford Nanopore (Oxford Nanopore Technologies, ONT, Oxford, UK) and PacBio (SMRT Technologies, Pacific Biosciences of California Inc., USA). These TGS technologies, under development since 2008, enable the generation of substantially longer reads—sometimes even spanning entire chromosomes—which significantly surpasses the capabilities of previous NGS technologies. Although error rates remain higher than those in NGS, with Illumina’s technology currently offering the most accurate sequencing method, TGS excels in numerous applications with a significantly lower sequencing depth and is rapidly gaining prominence in the field of DNA sequencing [106]. Amongst the advantages of TGS, and due to the long read sequences obtained, is the easy sequencing of the traditional and extremely challenging large repetitive genomic regions, large INDELs, CNVs, and SVs, which can be extremely beneficial in the comprehensive characterization of genomes under normal or pathological conditions [107]. TGS can obtain this information with minimal bias in GC- and AT-rich regions, which is a significant challenge for NGS due to its shorter read lengths. However, both PacBio and ONT still exhibit higher error rates than Illumina’s technology (10–15%), often necessitating correction of TGS data with NGS. As a result, the primary approach currently involves a combined TGS and NGS method, driving the rapid development of computational tools to support downstream analyses [49]. However, the TGS’s central role in the recent completion of the end-to-end mapping of the human project and many other genomes in the Telomere-to-Telomere (T2T) project [108,109,110,111] proved their versatility and applicability, which was after that applied in several studies regarding hematologic malignancies [112].

TGS, like NGS, is used not only for DNA but also for RNA material. The main difference is that NGS requires RNA to be converted into cDNA to prevent degradation and prepare it for sequencing. TGS, specifically Nanopore, enables the sequencing of native RNA, also known as direct RNA-seq, thereby avoiding RNA degradation and simplifying the process [113]. Also, Nanopore allows the easy identification of RNA modifications [114,115] enabling the identification of differential RNA modifications in MM samples with a novel computational method called xPore [116].

Another problem surpassed by TGS is the discovery of structural variants (SVs). These variants are traditionally assessed using Illumina’s technology in WES. However, as SVs are highly repetitive regions, the short-read sequences obtained by Illumina’s technology cause alignment errors and low signals, amongst other challenges [117,118], which different research groups have tried to correct through the development of computational tools [119,120,121,122]. TGS overcomes these challenges easily by generating long reads of 100 kb or more in length, which allows for the direct detection of most SVs with low library preparation techniques and smaller amounts of material [123,124,125,126]. Not only that, but the combinatory efforts of NGS, TGS, and other techniques have revealed very satisfactory results when trying to reconstruct complex SV events and unravel their different mechanisms and biological consequences [127].

## 3. Proteomics, Metabolomics, and Metagenomics Advances in the Study of MM

The industrialization of biology has led to the development of what is known as omics, a group of biological specialties that includes genomics, transcriptomics, epigenomics, proteomics, metabolomics, and metagenomics. Each omic aims to uncover in-depth knowledge by defining and quantifying pools of biological molecules, along with their structure, function, and dynamics. The previous chapters primarily covered advances in genomics (DNAseq), transcriptomics (RNAseq), and epigenomics (DNAseq); however, other omics have also produced significant findings in the field of MM. In this section, three additional omics and their impacts on MM are discussed.

Proteomics is a recent technology that has emerged as a valuable tool in cancer biology, aiding in prognosis and the identification of new therapeutic targets [128]. Specifically, blood-based targeted mass spectrometry in proteomics has proven to be a sensitive and minimally invasive alternative for measuring MM disease activity [129]. Combining several strategies is proving their efficacy; recently, a multiomics study involving deep tandem mass tag-based quantitative global (phosphor)proteomics, RNA-seq, and ONT sequencing of 138 patients with plasma cell malignancies encompassing MM and MGUS has revealed the potential of multiomics in the study of cancer, uncovering a prognostic protein signature and revealing the already well-known highly deregulated state of cells in plasma cell malignancies [130].

Metabolomics is a rapidly evolving omics science and a powerful tool to evaluate metabolism at a cellular or systemic level [131]. Targeted and non-targeted metabolomic analysis suggested that there is a significant metabolic signature change in the MM patients, with changes in amino acid metabolism as well as with the appearance of differential amino acid metabolite signatures associated with clinical indicators in the disease [132]. Results from a recent systematic review [133] revealed that the most impacted metabolic pathways are the citrate cycle, arginine and proline metabolism, D-glutamine/D-glutamate metabolism, histidine metabolism, and the urea cycle [132,134,135,136,137,138]. Interestingly, some MM cancer cells have a high demand for glutamine [139,140,141]; thus, glutamine uptake inhibition may prevent MM growth and may be associated with higher sensitivity to anti-MM drugs [142,143]. Other results suggest that the high activity of the serine–glycine one-carbon (SGOC) pathway is related to tumor resistance to chemotherapy [144], suggesting that blocking glycine uptake may be a promising therapeutic approach [145,146]. Also, amino acid levels like proline were related to osteolysis in MM [147,148,149], while others like leucine and valine have revealed diagnostic potentials [136]. Another combinatory study with proteomics suggested a lower concentration of PC lipids in MM [150]. Finally, and amongst the huge number of results in this field, obesity and aging stand out as known risk factors for MM, both being related to adipose tissue levels in the BM [151,152].

Finally, metagenomics is currently emerging as one of the most powerful techniques to measure microbial activity and composition, revealing underlying associations between the gut microbiome and several diseases, specifically MM [153,154]. Enrichment of nitrogen-recycling bacteria was highly correlated with the progression of MM [155]. Interestingly, it has been suggested that microbiota can also impact the immune response to tumor vaccination; thus, targeting gut microbes and tumor vaccines to remodel and modulate the microenvironment of the tumor could likely enhance anti-tumor immunity [156,157,158]. Dietary factors have been hypothesized to be associated with sustained MRD negativity and long-term survival in MM [159]. A short review of the aforementioned findings is summarized in Table 3.

## 4. Overall Aspects of Artificial Intelligence

AI is a broad scientific field focused on simulating human intelligence through the development of algorithms that enable learning from experience, reasoning, decision-making, and problem-solving—tasks traditionally performed only by human minds [160]. AI has expanded to encompass several subfields, such as ML and DL, and have emerged as powerful methodologies for extracting valuable information from data and problem resolution across diverse disciplines, including the healthcare system [161]. Based on their objectives, ML algorithms can be broadly categorized into three groups: supervised learning, unsupervised learning, and reinforcement learning, each offering distinct methodologies and applications.

Supervised ML (SML) involves training algorithms on labeled datasets, enabling them to learn the relationship between input features and the target output [160]. The algorithm undergoes iterative learning to predict the target variable based on the features, refining its predictions to effectively learn from the training data. In contrast, unsupervised ML (UML) operates with unlabeled data, meaning the dataset lacks predefined labels or categories [162]. UML uncovers hidden patterns or relationships within the data without explicit guidance, using techniques such as clustering and dimensionality reduction. For example, clustering analysis can be applied to patients with similar genetic signatures to identify common underlying causes [163]. Finally, reinforcement learning (RL) is an ML type in which an agent learns to make optimal decisions by interacting with an environment and receiving feedback through rewards or penalties [164]. An example of RL in healthcare is its application in personalized medicine for chronic diseases such as diabetes, where the agent optimizes patient treatment by continuously learning from health data and treatment responses [165]. Finally, DL (also part of the AI field) involves using advanced mathematical techniques to create “artificial neural networks” (ANN), systems inspired by the structure and function of neural networks in the human brain. These networks are composed of layers of nodes, or "neurons", connected to each other, where each connection has a weight that adjusts the influence of one neuron over another. Neural networks learn through a training process that involves adjusting these weights to minimize prediction error. By passing input data through multiple layers, each layer learning increasingly complex representations of the information, neural networks can identify intricate patterns in large volumes of data. Neural networks are particularly useful in tasks such as image recognition, natural language processing, and predicting complex outcomes in medical and genetic data, enabling researchers and clinicians to advance in the diagnosis and treatment of complex diseases, such as MM [166]. Table 4 shows a brief and comprehensive summary of the discussed AI methods.

### 4.1. Innovations in Multiple Myeloma Diagnosis Through Artificial Intelligence

AI holds immense promise in the diagnosis and treatment of MM, offering advanced techniques capable of analyzing large datasets—including genetic, imaging, and clinical data—to identify complex patterns and genomic alterations that may be undetectable through conventional methods. This capability is especially valuable in a complex and heterogeneous disease like MM. In this context, multiparametric flow cytometry (MFC) was applied to fresh bone marrow aspirates from 348 patients to differentiate between MM and MGUS. This approach identified differential expression markers between the two conditions, highlighting the importance of the CD27 and CD38 antigens, and produced a predictive classification algorithm with an accuracy of ≥95% [167]. Additionally, MoSaicNet and AwareNet deep learning methods were developed for analyzing BM trephine biopsies, achieving an AUC of >0.98 for tissue and cellular classification. These tools revealed that spatial heterogeneity, rather than cell density, differentiates MGUS from newly diagnosed multiple myeloma (NDMM), highlighting the reduced proximity of BLIMP1⁺ tumor cells to CD8⁺ T cells in MGUS. Post-treatment analysis showed a decrease in BLIMP1⁺ tumor cell density and changes in the immune microenvironment, underscoring the utility of DL in understanding MM marrow cellular architecture [168]. Since bone marrow biopsy and aspiration are procedures that may not always be ordered by physicians unless there is a strong suspicion of MM, the development of biomarkers in routine laboratory tests could be crucial for enabling rapid MM diagnosis. To this end, blood samples were collected from multiple hospitals to develop an innovative model for diagnosing MM. This model, based on demographic features and routine blood biomarkers, utilized the AdaBoost-DecisionTable algorithm. Despite certain limitations, such as incomplete clinical data and a small validation dataset, the model achieved high accuracy and a strong area under the curve (AUC), both key metrics for assessing its potential clinical applicability [169]. Additionally, an AI-assisted diagnosis system analyzed 4187 blood and biochemical records from Shengjing Hospital (1741 MM, 2446 non-MM) using hemoglobin, serum creatinine, serum calcium, immunoglobulins (A, G, M), albumin, total protein, and albumin-to-globulin ratio. The Gradient Boosting Decision Tree (GBDT) algorithm achieved the highest precision for accurately diagnosing MM [170].

Matrix-assisted laser desorption/ionization and time-of-flight mass spectrometry (MALDI-TOF MS) is a powerful and highly sensitive tool for detecting large numbers of peptides and proteins in serum, making it a robust clinical diagnostic method [171]. Deulofeu et al. utilized this technology to acquire low-mass profiles of peripheral blood plasma from MM patients and healthy donors, which served as inputs for artificial neural network (ANN) algorithms that effectively classify MM patients, offering a minimally invasive approach to MM diagnosis [166]. In a subsequent study by the same group, proteins in peripheral blood plasma were precipitated using a two-step extraction protocol to improve MALDI-TOF MS resolution. Machine learning algorithms combined with MALDI-TOF MS enabled accurate classification and discrimination between two monoclonal gammopathies, MM and PCL, achieving an accuracy of 71.5% under 10× repeated 5-fold cross-validation [172].

The detection of M-spike protein levels—immunoglobulins overproduced by the proliferating malignant MM clone—is performed using serum protein electrophoresis (SPEP), which requires skilled personnel and specialized equipment. By integrating readily available clinical and laboratory data with a random forest (RF) ML algorithm, M-spike values were determined and found to correlate highly with those obtained through conventional laboratory methods [173]. While the ML algorithm showed a strong correlation with M-spike values, incorporating additional factors, such as chromosomal translocations or tumor genomics, along with a larger patient dataset, could further improve performance. In a related study, Sopasakis et al. compared various ML algorithms for M-protein identification using numerical data from serum protein capillary electrophoresis and found that decision tree algorithms could detect the presence of M protein with high accuracy [174]. Natural language processing (NLP) was applied to SPEP reports to detect monoclonal gammopathy, achieving high accuracy across multiple hospitals, with ML models outperforming rules-based methods, particularly when models were applied across sites [175]. Employing ML methods as a support tool can assist in minimizing the number of unnecessary follow-up analyses, which are frequently conducted due to uncertainty or lack of experience by the individual interpreting the initial electrophoresis results.

A critical aspect of MM diagnosis is the analysis of bone marrow (BM) aspirates from patients, a manual procedure that is time-consuming and subject to considerable inter- and intra-observer variability. A study by Chandradevan et al. presented a digital prototype based on convolutional neural networks (CNNs), a deep learning (DL) technique, which demonstrated higher accuracy in detecting and classifying both non-neoplastic and neoplastic cells in MM BM aspirates [176]. However, the authors noted some limitations, including the exclusion of disease cases in the training data, small sample size, and untested performance on larger, denser regions, as the study focused on areas with clear, well-preserved cell structures.

In another study, Rasal et al. proposed a neural network based on improved empirical mode decomposition (IEMD), also a DL approach, to recognize MM cell nuclei and distinguish cell membranes, specifically the cytoplasm. They further introduced a novel counting algorithm for MM cells segmented from images and validated the methodology with publicly available datasets, highlighting the potential of this innovative image segmentation method for early MM diagnosis [177]. Additionally, a technique based on generative adversarial networks (GAN), called MultiPathGAN, was applied to BM microscopic images from various databases to standardize stain styles and augment data, enhancing the model’s adaptability to different staining techniques. Following standardization, the development of MobileViTv2—a hybrid model combining CNNs and vision transformers (ViT)—enabled efficient diagnosis of hematologic malignancies, including MM [178].

Further, an AI-powered platform called Morphogo analyzed 305,019 cell images from BM and peripheral blood (PB) smears for cell morphology screening of MM cells, a task conventionally performed by expert technicians. Morphogo demonstrated high accuracy, sensitivity, and specificity in detecting circulating plasma cells in PB smears, outperforming manual microscopy [179]. Implementing AI-powered computer-assisted diagnostic systems can significantly enhance efficiency and support pathologists in the MM diagnostic process. This approach would help reduce time, costs, and reliance on expert evaluation.

The incorporation of genetic features for MM diagnosis may also optimize treatment strategies. In this context, a new ANN classification model was developed to diagnose and assess high-risk status in MM patients, using features such as age, gender, percentage of BM plasma cells, white blood cell (WBC) count, and cytogenetic alterations analyzed by FISH. Tested on 477 cases, the algorithm achieved 94% accuracy, with preliminary results identifying a correlation between the percentage of BM plasma cells, WBC, and genetic risk factors in MM diagnosis [180].

To provide comprehensive insights into the extent of BM infiltration in MM patients, whole-body 18F-fluorodeoxyglucose (FDG) positron emission tomography/computed tomography (PET/CT) has emerged as an imaging technology that combines morphological and anatomical findings with functional metabolic activity [181]. While this promising technology may encounter variations in scan interpretation due to diverse infiltration patterns in the BM, the integration of AI could effectively address these discrepancies and improve diagnostic accuracy. A study carried out by Satoh et al. aimed to establish a standard for BM FDG uptake using a deep learning-based organ segmentation method on PET/CT images of 98 healthy adults [182]. In another study, the researchers aimed to validate a novel deep learning-based tool that automates the assessment of BM metabolism in MM patients using whole-body PET/CT images. The study analyzed PET/CT scans from 35 untreated MM patients, and the automated method showed a strong positive correlation with traditional visual PET/CT analysis and clinical data, suggesting it could be a reliable tool for standardizing PET/CT interpretation in MM [183].

ML-based CT texture analysis effectively differentiates MM from osteolytic metastatic bone lesions, achieving an accuracy of 78.8–92.3%, with the k-nearest neighbors model performing the best [184]. Furthermore, machine learning-based CT utilizing photon-counting detectors significantly enhances the visibility of multiple myeloma lesions compared to energy-integrating detector CT, leading to improved detection of lytic lesions and associated abnormalities [185]. Together, these advancements highlight the potential of ML techniques in enhancing diagnostic accuracy and visibility in the evaluation of MM.

AI techniques enable the analysis of large and complex datasets (e.g., genetic, imaging, clinical) to detect underlying patterns and genomic alterations not identifiable by conventional methods. Applications such as flow cytometry, mass spectrometry, and PET/CT imaging, combined with AI, have shown high diagnostic accuracy in distinguishing MM from related conditions. Although limitations exist, such as small validation datasets, AI-driven approaches are poised to improve diagnostic efficiency, reduce costs, and support personalized treatment strategies in MM. Additionally, AI holds promise as a valuable tool to assist specialized personnel, enhancing decision-making and reducing reliance on manual evaluations.

A short summary of selected examples of the aforementioned findings is presented in Table 5.

### 4.2. Prognosis of Multiple Myeloma: Advances Through Artificial Intelligence

#### 4.2.1. Advancements in Risk Stratification

Clinical prediction tools for MM remain limited. The International Staging System (ISS) is a widely used prognostic tool for risk stratification in newly diagnosed MM patients based on β2-microglobulin and albumin levels. It classifies patients into three groups with varying overall survival rates. In 2015, the Revised ISS (R-ISS) was introduced, adding lactate dehydrogenase (LDH) and high-risk cytogenetic abnormalities to improve accuracy. However, both ISS and R-ISS have limitations, as they fail to fully stratify certain patients, particularly within low- and intermediate-risk groups.

While predictive biomarkers help guide therapy selection, there is still insufficient data to routinely tailor treatment strategies for MM based on risk level. Emerging technologies, such as AI, can enhance patient stratification by analyzing complex clinical and laboratory data, potentially improving the selection of initial treatments, intensifying therapy for high-risk patients, and reducing treatment for low-risk cases. Developing AI-powered, accessible staging models that use simple clinical and lab parameters would optimize healthcare resources and reserve molecular testing for patients who relapse or require targeted therapies in clinical trials. In this regard, a novel risk stratification system for MM, known as the Modified Risk Staging (MRS) system, was introduced by Farswan et al. This system leverages machine learning (ML) and six basic laboratory parameters: albumin, β2-microglobulin (β2M), calcium, eGFR, hemoglobin, and age. Trained and validated on newly diagnosed MM patient data, the model offers a cost-effective alternative to genomic testing. Its accuracy, confirmed through cross-validation and ROC analysis, enhances risk group classification and survival predictions, particularly for high-risk patients [186].

Recently, an unsupervised machine learning (UML) model was developed for risk stratification in MM, integrating clinical, biochemical, and cytogenetic data from patients treated in clinical trials by the Spanish Myeloma Group [187]. This model demonstrates improved prognostic accuracy, particularly for patients within the intermediate-risk R-ISS 2 group, surpassing the predictive power of traditional ISS and R-ISS scores. It identifies two distinct patient clusters with significantly different survival outcomes, highlighting the potential of combining existing staging systems with machine learning techniques to enhance MM risk stratification [187]. The combination of gene expression profile (GEP) and clinical data, using the GuanRank hazard ranking model with Gaussian process regression (GPR), led to the identification of new gene signatures associated with MM progression. While the integration of clinical data improved the model’s performance, the study emphasizes that GEP alone is insufficient for precise prognostication. The authors suggest that incorporating higher-quality cytogenetic data could further enhance the model’s predictive accuracy [188]. Orgueira et al. developed a machine learning model (IAC-50) integrating clinical, biochemical, and gene expression data from the CoMMpass cohort to predict overall survival (OS) and optimal drug combinations in MM. The model, which includes 50 variables such as patient age, ISS stage, β2-microglobulin, and 46 gene expressions, showed promise in high-risk cytogenetic patients, providing personalized treatment predictions and potentially outperforming traditional cytogenetic risk stratification [189]. Additionally, an advanced Graph Convolutional Network-based Risk Stratification system (GCRS) has been introduced for predicting cancer risk stages in newly diagnosed MM patients. By combining multiple connectivity graphs derived from clinical and laboratory data, GCRS effectively classifies patients into low, intermediate, and high-risk groups [190].

#### 4.2.2. Integrating Imaging Data and AI for Improved Risk Stratification in MM

Current prognostic models for MM primarily rely on blood and BM samples, with few models integrating imaging data due to the complexities of manual image assessment. An AI convolutional autoencoder was used to extract features from FDG PET/CT images in MM patients, with the goal of risk stratification [191]. By compressing the image data, the AI algorithm successfully identified feature clusters that enabled predictions of progression-free survival (PFS). Both supervised and unsupervised clustering methods produced three distinct patient clusters, with cluster C indicating poorer PFS. However, the analysis was limited by PET images being restricted to the torso, excluding critical areas where osseous MM lesions often occur. Despite this, FDG PET/CT imaging showed that tumor burden indicators, including metabolic tumor volume (MTV) and total lesion glycolysis (TLG), are significant predictors of patient prognosis. This AI-based clustering approach shows potential for enhancing prognostic accuracy in MM patients [191]. In another model, PET-based features like MTV, CT-based features, and clinical parameters were identified as significant predictors of PFS. Six ML algorithms, including Cox models and gradient boosting, were evaluated, revealing that models integrating PET, CT, and clinical data significantly outperformed those based solely on clinical parameters [192]. Schenone et al. demonstrated the potential of an AI-based approach using CT data for the automatic stratification of transplanted MM patients into relapsed and non-relapsed groups, as well as for identifying radiological biomarkers with prognostic value. The study included 51 transplanted patients, 33 (64%) of whom presented with focal lesions, using fuzzy clustering (FC) and Hough transform filtering (HTF) for patient stratification. Despite limitations, including the use of a single radiomics tool and a relatively small patient sample, this study highlights the potential for image-based prognostic biomarkers in MM follow-up [193]. Utilizing a DL-based segmentation approach on CT images, the presence of sarcopenia was assessed in 322 patients with newly diagnosed MM, revealing that 53% were sarcopenic. Sarcopenic patients exhibited significantly worse outcomes, with a median overall survival of 44 months and a 2-year mortality rate of 40%, compared to 90 months and 18% for non-sarcopenic patients, indicating the prognostic value of sarcopenia in this population [194].

Similarly, applying AI to magnetic resonance imaging (MRI) data for multiple myeloma (MM) prognosis could prove beneficial, eliminating the need for blood and bone marrow samples. Using 3D convolutional neural networks (CNNs) and Gradient-weighted Class Activation Mapping (Grad-CAM) to analyze whole-body diffusion-weighted MRI data, a recent study introduced a novel prognostic model for MM [195]. A retrospective analysis of 142 patients revealed significant differences in progression-free survival (PFS) between good and poor prognostic groups, with key prognostic factors identified as MRI signals from the spleen and vertebral bones. This AI model, validated externally, demonstrated predictive accuracy comparable to existing models like the International Staging System (ISS) and Revised International Staging System (R-ISS), underscoring AI’s potential for prognosis prediction in MM using only MRI data. However, challenges such as small sample size and treatment variability require further research [195]. A separate retrospective study aimed to develop and validate an MRI-based radiomics model for predicting high-risk cytogenetic status (HRC) in 89 MM patients [196]. Six classifiers were tested, with the logistic regression (LR) model demonstrating superior performance (AUC: 0.82; sensitivity: 84.1%; specificity: 68.1%) compared to others. The two-sequence MRI models, T1-weighted (T1W) and fat-suppression T2-weighted (FS-T2W), outperformed single-sequence models in distinguishing HRC from non-HRC statuses [196]. In another study by the same group, a radiomics approach based on spinal MRI was used to predict high-risk cytogenetic abnormalities (HRCAs) in MM patients [197]. Analyzing 248 lesions (111 HRCA and 137 non-HRCA), the top nine radiomic features were selected through LR to create a radiomics model, and a combined model incorporating clinical characteristics was also developed. The radiomics model showed a sensitivity of 0.789, specificity of 0.787, and accuracy of 0.788, highlighting its potential as an independent tool for predicting HRCAs in MM patients using routine spinal MRI. Additionally, this model can identify patients likely to have HRCAs, thereby recommending further genetic examination to improve treatment and prognosis. Future multicenter studies with external validation and additional clinical data could enhance the predictive performance of radiomics models.

#### 4.2.3. Predictive Modeling for Treatment Responses

Predicting clinical drug response in cancer treatment is essential for improving patient outcomes and reducing costs. Given the challenges of analyzing large datasets generated by high-throughput drug screening, advanced ML algorithms are necessary for making accurate predictions of drug sensitivity [198]. In this context, GEP databases linked to clinical chemotherapy responses and suitable for ML applications could prove highly valuable for predicting chemotherapy outcomes [199]. Povoa et al. introduced the Multi-Learning Training (MuLT) approach, which combines supervised, unsupervised, and self-supervised ML algorithms to predict treatment sensitivity (TS) in MM patients. By incorporating gene expression data that reflects genetic abnormalities detected by FISH testing, MuLT enhances TS prediction accuracy, achieving an AUC of 68.70% in cross-validation experiments. Applied to data from 1525 newly diagnosed MM patients from the MMRF CoMMpass study, the method identified alternative first-line treatment options for 17.07% of patients, demonstrating its potential for improved treatment stratification [200]. Indeed, new RNA sequencing profiles were generated for 53 MM patients treated with two chemotherapy regimens involving bortezomib: PAD (bortezomib, doxorubicin, dexamethasone) and VCD (bortezomib, cyclophosphamide, dexamethasone). Using five ML methods, classifiers were developed to differentiate between good and poor responders, with five genes (FGFR3, MAF, IGHA2, IGHV1-69, and GRB14) found to be upregulated in good responders. The binomial naïve Bayes (BNB) model achieved the best performance for the PAD + VCD cohort (AUC 0.84), while the multi-layer perceptron (MLP) model excelled in the VCD cohort (AUC 0.89), highlighting the potential of AI models combined with RNA sequencing profiles to identify patient-specific responses to chemotherapy [199]. A machine learning method called simulated treatment learned signatures (STLsig) identified gene signatures that predict which MM patients are likely to benefit from proteasome inhibitors such as bortezomib and carfilzomib. In a study of 910 patients, STLsig successfully differentiated responders, potentially improving treatment decisions and providing insights into treatment mechanisms [201]. The ubiquitin–proteasome pathway risk score (UPPRS), derived from nine ubiquitin proteasome pathway-associated genes, effectively predicts overall survival in MM patients and identifies those likely to benefit from proteasome inhibitors; ML models integrating UPPRS and the ISS enhance survival predictions [202]. Additionally, research conducted by Kropivsek et al., using advanced methods like multiplexed immunofluorescence and deep-learning-based single-cell phenotyping, identified key molecular pathways associated with drug sensitivity, offering critical insights for personalized treatment approaches in MM [203].

#### 4.2.4. Minimal Residual Disease (MRD) Prediction

MRD in MM refers to the small number of cancer cells remaining after treatment, undetectable by standard methods. Advanced techniques like next-generation flow cytometry (NGF) and sequencing (NGS) allow for highly sensitive MRD detection. Achieving MRD negativity is linked to better progression-free survival (PFS) and overall survival (OS), making it a key prognostic marker in various stages of MM. Guerrero et al. developed an ML model to predict MRD outcomes in MM integrating key genetic factors, including t(4;14) and del(17p13), along with tumor burden markers and immune-related biomarkers. By assigning weighted importance to these variables, the model accurately predicted MRD status in 71–72% of patients in both training and validation cohorts. Additionally, it identified a subgroup of patients with exceptional progression-free and overall survival rates at 5 years. This ML model offers a novel approach to personalizing treatment, enabling early MRD prediction and guiding therapy adjustments [204].

A short summary of selected examples of the aforementioned findings related to prognosis, patients’ stratification, and treatment modeling using the AI approach is presented in Table 6.

## 5. Conclusions and Future Perspectives

MM remains a complex and challenging malignancy despite significant advancements in its diagnosis and treatment. Key fronts include early diagnosis, disease staging, and treatment response, which are essential for improving patient outcomes. Although modern drug combinations have extended survival, many patients relapse, indicating the need for continuous innovation. Traditional diagnostic techniques like FISH and karyotyping have limitations in accuracy, emphasizing the need for more precise tools.

Emerging technologies, particularly AI and omics, including high-throughput sequencing (NGS and TGS), offer new opportunities to improve disease characterization and treatment. However, integrating these technologies into clinical practice has been slow due to the complexity of MM, computational demands, high costs, and the ever-existent gap between experimental and computational sciences.

The use of NGS in research has led to the discovery of several novel genomic markers, structural variations, and somatic mutations associated with MM, underscoring the utility of this method for characterizing the disease at a deeper molecular level. The inclusion of NGS in the study of DNA (DNAseq, specifically targeted NGS panels [48], RNA (RNAseq), and the epigenetic landscapes of MM have identified critical metabolic pathways, therapeutic targets such as BCMA and SLAMF7 [32,66], roles of DNA hypo-/hypermethylation related to MM outcomes [78,79], histone modifications, and microRNA expression linked to MM progression [53,73]. Combined DNAseq and RNAseq approaches have also proven valuable for assessing disease progression and treatment outcomes, with liquid biopsy approaches emerging as a less invasive method for genomic profiling [32,71].

In recent years, technological advances have led to the development of TGS technologies, which offer longer sequencing reads, enabling the characterization of challenging genomic regions and the direct sequencing of native RNA [107,113]. These advances have been essential in addressing limitations associated with NGS short-read sequencing. However, despite the strengths of TGS, it still has higher error rates than Illumina’s technology, the dominant NGS method. This makes the combined use of NGS and TGS crucial for overcoming various high-throughput sequencing challenges and is becoming a common approach in large-scale genomic studies, significantly advancing research in hematologic malignancies like MM [49,127].

Omics fields such as proteomics, metabolomics, and metagenomics aim to fully characterize the biomolecular landscape of MM. Each field presents unique challenges in data analysis and interpretation, yet they provide valuable insights into biomarkers, therapeutic targets, and the role of the microbiome in MM progression [130,133,155]. However, translating these findings into clinical practice will require overcoming technical and validation challenges.

The integration of complex datasets, comprising genetic, imaging, and clinical information, by AI, has revealed substantial potential to enhance the diagnosis of MM, allowing for the identification of patterns that conventional approaches may fail to recognize. Different studies reviewed in this work demonstrate significant advancements, particularly in distinguishing MM from related conditions using ML algorithms. Furthermore, AI can enhance prognostic capabilities by analyzing clinical data and identifying risk factors, potentially allowing for more tailored treatment plans based on individual patient profiles. In addition, AI shows promising potential in drug discovery for MM [205]. AI-driven analyses of large-scale multiomics datasets could offer the possibility of identifying novel drug targets and predicting therapeutic efficacy, which could significantly accelerate the development of new treatments, though further research and validation are needed to fully realize its impact in this area.

Despite these promising outcomes, the limitations of current AI-based approaches cannot be overlooked. Many models, while demonstrating high accuracy, are constrained by small validation datasets, incomplete clinical data, and limited context applicability. For instance, several diagnostic models relied on BM biopsy samples, which are invasive and may not always be available. In contrast, imaging techniques such as MRI and PET-CT offer non-invasive alternatives that can assist in both diagnosis and prognosis by providing detailed visualization of bone lesions and soft tissue involvement, reducing the need for invasive procedures while still delivering crucial clinical information. Additionally, while some algorithms perform well in structured datasets, their performance in real-world, heterogeneous populations remains to be fully validated. Furthermore, challenges such as variations in staining techniques, sample sizes, and the exclusion of complex cases from training datasets limit the clinical application of these models.

In response to these limitations, it is essential to emphasize that the efficacy of AI in MM diagnostics hinges significantly on the availability of high-quality, validated, and reproducible datasets. Current AI models often face challenges due to incomplete or inconsistently labeled data, limiting their reproducibility and clinical relevance. To address this, a robust focus on research dedicated to curating comprehensive and standardized datasets is imperative. Such datasets, appropriately labeled and validated, would enhance the reliability of AI models, enabling more accurate and clinically applicable diagnostic tools. Addressing these limitations through larger, multicenter studies and integrating more diverse clinical and genomic factors will be essential for refining AI-driven diagnostic tools. Nevertheless, AI holds considerable promise in improving diagnostic and prognostic accuracy, advancing drug discovery, and supporting personalized treatment strategies for MM, underscoring the need for ongoing development and validation efforts.

MM’s complexity, marked by diverse genetic, phenotypic, and clinical manifestations that often overlap with other plasma cell disorders, presents considerable challenges for accurate and timely diagnosis. Early detection is essential for improving patient outcomes; however, the insidious nature of MM frequently results in misdiagnosis or delays until the disease reaches a more advanced stage. These novel technologies, omics –with the inclusion of high-throughput sequencing- and AI, despite their limitations, offer promising strategies for the future development of precision medicine in the diagnosis, prognosis, and improvement of treatment of MM.

## Figures and Tables

**Table 1:** Main genomic alteration observed in MM.

Genomic Alteration	Prognostic Implication	Detection Method
t(11;14)(q13;q32)	Intermediate prognosis, good response to treatment.	FISH, NGS, RT-PCR
IgL locus	Poor prognosis, associated with reduced survival.	FISH, SNP arrays
MYC/RAS overexpression	Associated with progression from MGUS to MM.	FISH, NGS
Del(17p13)	Poor prognosis, hypodiploidy, resistance to treatment, and lower survival rate.	FISH, NGS
Gain of 1q (Amp(1q))	High-risk marker, associated with increased relapse rates.	FISH, NGS
t(4;14) translocation	Intermediate/poor prognosis, therapeutic resistance.	FISH, NGS
Lower frequency translocations (t(14;16)(q32;q23), t(14;20)(q32;q11) (2%) y t(8;14)(q24.3;q32))	Poor prognosis, aggressive disease.	FISH, NGS
Hyperdiploidy	Better prognosis.	Conventional karyotyping, FISH
Hypodiploidy	Poor prognosis, associated with chromosomal losses.	FISH, SNP arrays, NGS

**Table 3:** Omics approaches in MM.

Omic Approach	Key Findings	Clinical Applications	Technology [Reference]
Genomics (NGS)	Identification of mutations such as IRF4, EGR1, del(17p), and t(4;14). Identification of genetic loci related to bortezomib-induced peripheral neuropathy.	Personalized treatment strategies, risk stratification, and treatment optimization.	Whole-genome sequencing (WGS), targeted sequencing [51,58,63]
Transcriptomics (RNA-seq)	Gene expression profiling (e.g., BCMA, SLAMF7); alternative splicing analysis.	Target discovery, understanding of MM clonal evolution, non-invasive monitoring.	RNA sequencing (RNA-seq), single-cell RNA-seq [32,66]
Epigenomics	DNA methylation patterns, histone modifications associated with tumor progression, and miRNA signatures.	Prognostic biomarkers, potential therapeutic interventions targeting epigenetic modifications.	DNA methylation arrays, ChIP-seq [73,77,78]
Proteomics	Protein signature/biomarkers for disease activity and progression, protein signatures for prognosis.	Non-invasive monitoring using blood tests, identifying therapeutic targets.	Mass spectrometry (MS), protein arrays, and combined techniques [128,130]
Metabolomics	Alterations in amino acid metabolism, changes in the citrate cycle, arginine and proline metabolism, D-glutamine/D-glutamate metabolism, histidine metabolism, and urea cycle.	Therapeutic targets (e.g., glutamine inhibition).	NMR spectroscopy and mass spectrometry [133,139,144]
Metagenomics	Links between gut microbiota and MM progression, potential immune modulation.	Microbiome-based interventions; enhanced understanding of disease mechanisms.	Next-Generation Sequencing (NGS), 16S rRNA sequencing [153,155,156]

**Table 4:** Summary of general AI Approaches in Healthcare.

Method	Description	Examples
AI	Simulation of human intelligence through algorithms, including learning, reasoning, and decision-making.	Applied in healthcare for diagnosis, predictive analytics, and personalized treatment [160,161].
ML	Subfield of AI for data analysis and problem-solving includes supervised, unsupervised, and RL.	Used in medical diagnostics, genomics, and drug discovery.
SML	Uses labeled data to train algorithms to predict target outcomes based on known input-output relationships.	Predictive models for disease outcomes, such as identifying cancer risk based on patient data [160].
UML	Analyzes unlabeled data to discover hidden patterns or clusters without predefined labels.	Clustering genetic data to identify groups of patients with similar disease traits improves diagnosis and treatment [162,163].
RL	An agent learns optimal decision-making through feedback (rewards/penalties) from interactions with the environment.	Used in personalized medicine, such as adjusting diabetes treatments based on patient response data [164,165].
DL	Creation of ANN inspired by the human brain structure. Passes data through layers to identify complex patterns.	Tasks like image recognition, natural language processing, and predicting medical outcomes, advancing diagnosis and treatment of complex diseases like MM [166].

**Table 5:** Applied AI approaches in MM diagnosis.

AI Application Area	Selected Example	Clinical Applications	Technology [Reference]
AdaBoost-DecisionTable Model	Development of an innovative model using demographic and routine blood biomarkers; achieved high accuracy.	Rapid MM diagnosis based on readily available clinical and laboratory data from multiple hospitals.	AdaBoost-DecisionTable algorithm [169]
Gradient Boosting Decision Tree (GBDT)	High precision in diagnosing MM based on biochemical records (e.g., hemoglobin, serum calcium, albumin).	Supports accurate MM diagnosis by analyzing biochemical markers, reducing reliance on invasive procedures.	GBDT algorithm [170]
MoSaicNet and AwareNet	DL methods for analyzing BM trephine biopsies. Spatial heterogeneity differentiates MGUS from NDMM, highlighting reduced proximity of BLIMP1⁺ tumor cells to CD8⁺ T cells in MGUS.	Differentiation of MM and MGUS.	MoSaicNet and AwareNet [168]
Random Forest (RF) for M-spike detection	Integrates clinical data to determine M-spike levels, showing correlation with conventional methods.	Supports detection of M-spike protein levels in MM without the need for specialized equipment, minimizing follow-up analyses.	RF algorithm [173]
Convolutional Neural Networks (CNN)	Digital prototype using CNNs for detecting non-neoplastic and neoplastic cells in BM aspirates; high accuracy in cell classification.	Reduces manual work in BM analysis, assisting pathologists in MM diagnosis.	CNN [176]
ANN Classification Model	Uses genetic and clinical features to assess high-risk status in MM with 94% accuracy.	Uses genetic and clinical features to assess high-risk status in MM with 94% accuracy [178].	ANN model [180]
Whole-Body Imaging Analysis	AI tool correlates well with traditional PET/CT analysis, offering consistent di-agnostic interpretation in MM.	Standardizes PET/CT for assessing BM metabolism in MM patients.	DL + PET/CT [183]

**Table 6:** AI approaches in MM prognosis, stratification, and treatment.

AI Application Area	Key Features	Clinical Applications	Technology [Reference]
Unsupervised ML Model for Risk Stratification	Integrates clinical, biochemical, and cytogenetic data; improves accuracy in R-ISS 2 intermediate-risk group.	Identifies patient clusters with different survival outcomes, enhancing risk stratification.	UML integrating clinical, biochemical, and cytogenetic data [186]
GEP and Clinical Data Model	Combines GEP with clinical data to identify gene signatures for MM progression; suggests adding cytogenetic data.	Provides insights into MM progression, suggesting treatment adjustments.	GEP, GuanRank with Gaussian process regression [188]
IAC-50 Model	Integrates clinical, biochemical, and gene expression data from the CoMMpass cohort for personalized treatment.	Predicts overall survival and optimal drug combinations, aiding personalized treatments.	ML model from CoMMpass cohort data [189]
AI Convolutional Autoencoder for PET/CT Imaging	Extracts feature clusters from PET/CT for progression-free survival prediction, limited by torso-only scans.	Supports MM prognosis by predicting progression-free survival (PFS).	AI-based PET/CT analysis [187]
3D CNN and Grad-CAM for MRI Data.	Analyzes MRI signals from spleen and vertebral bones for PFS prediction; requires further research.	Predicts PFS solely from MRI data, offering a non-invasive prognosis tool.	3D CNN, Grad-CAM for MRI [195]
Simulated Treatment Learned Signatures (STLsig).	Identifies gene signatures predicting benefit from proteasome inhibitors; improves treatment decisions.	Supports targeted treatments by identifying responder patients.	STLsig for proteasome inhibitor response [201]
ML Model for MRD Prediction.	Predicts MRD based on genetic factors and tumor markers; achieved 71–72% accuracy in prediction.	Predicts MRD status and guides therapy adjustments for MM patients.	ML model integrating genetic and tumor burden data [204]
